# Supplementary material for: Practice, governance, and culture characteristics of lived experience organisations, and evidence of efficacy: A scoping review protocol
Source: PLoS One. 2023 May 5;18(5):e0283178. doi: 10.1371/journal.pone.0283178 (PMC10162514; doi:10.1371/journal.pone.0283178)
Supplement: S2 File — (DOCX) [file pone.0283178.s002.docx]

**S2 File. Published MEDLINE database search strategy (searched July 26, 2022).**

| **#** | **Search Strategy/String** | **Contextual Narrative** |
| --- | --- | --- |
|  | 1. "expert* by experience”.mp 2. ((peer* or consumer* or care* or “lived experience” or “living experience”) adj3 (workforce or work* or support* or advocate* or consult* or leader* or educator*)).mp. 3. 1 or 2 or 3 4. Mental health/ or mental health services/ or mental disorders/ 5. ("mental health” or “mental* ill*” or “mental disorder” or “behavio?ral health” or “psychosocial disability”).mp. 6. 5 or 6 7. (organi?ation* or workplace or workforce or management or service or corporate). mp. 8. Culture or Value* or Ethic* or Standard* or Guideline* or Principle* or “Decision making” or Management or Governance or Membership or Communicat* or Collaborat* or Support* or Develop* or Consult* or “professional development” or Train* or Qualif* or Skill* or Certif* or Evaluat* or “career*pathway” or “career progression” or Policy or Procedure or Advoca* or Quality assurance or Review or Mentor* or Supervision or Accountab* or Trauma-informed or Best-practi?e).mp. 9. 4 and 7 and 8 and 9 10. Limit 10 to (English language and yr=“2000 – Current”)) | Lines 1 and 2 are the terms relevant to participant.  Lines 4 and 5 are the terms pertaining to participant characteristics.  Line 7 are terms related to organization.  Line 8 are terms related to organizational outcomes.  Lines 3, 6, 7 and 8 use the ‘or’ function which means the returned result must include only one word/phrase from any of the specified lines.  Line 4 utilize relevant MeSH terms specific to the MEDLINE database. MeSH terms use a controlled vocabulary to group together articles on a similar topic. In cases where a subject heading has ‘exp’ written in front of it, this means the term is exploded during the search and associated terms that fall under that key word will also form part of the search.  Lines 1, 2, 4, 7 and 8 use truncation (an asterisk (*)), which represents any letter or combination of letters that may be used in its place. For example, the term parent* will search for parent**s** as well as parenting.  Lines 1, 2, 5 and 8 use quotation marks around words in each of these lines. Quotation marks mean that the phrase is searched rather than the separate words, and relevant terms will be identified with or without a hyphen. For example, “Best-practi?e” will be identified if it is written as best-practice as well as best practice.  Line 2 uses proximity searching, indicated by adj3. This means that all terms in the first bracket set, must appear within three words of the second bracket set in any order. For example, a reference containing peer advocate in any relevant field, would be identified.  Line 7 and 8 uses a ? to replace a character within the word. For example, behavio?ral will search for references containing either behavioural or behavioral.  Line 9 combines search terms for participant (line 3) AND participant characteristic (line 6) AND organization (line 7) AND organizational outcomes (line 8). This will identify studies focusing on all four components of the search strategy across the title, abstract, heading word, table of contents, key concepts, original title, tests & measures, MeSH word. Searching each of these fields is denoted by .mp.  Line 10 restricts all returned studies to those written in the English language and published from 2000 to the date of search. |

*Note*: MeSH = Medical Subject Heading; MeSH terms ‘peer assistance programs’ and ‘behavioural and mental disorders’ are only available in CINAHL and ‘peer counselling’ is only available in CINAHL, EMBASE, and PsycINFO. Therefore, these terms are not included in this table as it is a MEDLINE specific database search example.
