# Supplementary material for: Practice, governance, and culture characteristics of lived experience organisations, and evidence of efficacy: A scoping review protocol
Source: PLoS One. 2023 May 5;18(5):e0283178. doi: 10.1371/journal.pone.0283178 (PMC10162514; doi:10.1371/journal.pone.0283178)
Supplement: S3 File — (DOCX) [file pone.0283178.s003.docx]

**S3 File. Unpublished and grey literature search strategy.**

# Strategy 1. Targeted Website Browsing/Searching (i.e., search 1 website at a time)

Browse and/or search (using a variety of identified words) the websites of organizations (government, health organizations, NGOs, universities, research centres, etc.) that publish documents relevant to research question.

## Website Browsing Documentation:

| **Date** | **Organization name**  *E.g., VMIAC* | **URL**  *E.g.* [*https://www.vmiac.org.au/*](https://www.vmiac.org.au/) | **# of items screened (uploaded to citation management software)**  *E.g., 1* |
| --- | --- | --- | --- |
|  |  |  |  |
|  |  |  |  |
|  |  |  |  |

# Strategy 2. Search Engine Searching (Google.com)

1. All results on the first 15 pages will be clicked and screened for relevance
2. Search with various word combinations using Google, Google Scholar, and Advanced Google

## Documentation:

| **Date** | **Search engine**  *E.g., Google.com* | **Search strategy(s) including how items were selected**  *E.g., Search strategies:*   1. *lived experience peer support work organization practice* 2. *lived experience peer support work policy* 3. *lived experience peer support work governance* *Selection: Items were selected by scanning the first 15 pages from each search.* | **# of items screened (uploaded to citation management software)**  *E.g., Items Screened:*   1. *3* 2. *2* 3. *0* |
| --- | --- | --- | --- |
|  | *E.g., Google.com* | *E.g., Used* ***Advanced Google*** *site/domain search*  *Search strategies:*  *lived experience peer support work organization practice* ***site:gov***  *Selection: Items were selected by scanning the first 15 pages from each search* |  |
|  | *E.g., scholar.google.com* | *Search strategies:*  *lived experience peer support work organization practice*  *Selection: Items were selected by scanning the first 15 pages from each search* |  |

# Strategy 3. Grey Literature Database Search:

1. ProQuest Dissertations and Theses (Global)

This template was adapted from UBC Library (<https://guides.library.ubc.ca/ld.php?content_id=35738266>) and is based on the methods outlined in: Godin, K., Stapleton, J., Kirkpatrick, S. I., Hanning, R. M., & Leatherdale, S. T. (2015). Applying systematic review search methods to the grey literature: a case study examining guidelines for school-based breakfast programs in Canada. Systematic reviews, 4(1), 138. [DOI: 10.1186/s13643-015-0125-0](https://systematicreviewsjournal.biomedcentral.com/articles/10.1186/s13643-015-0125-0)
