# Supplementary material for: Practice, governance, and culture characteristics of lived experience organisations, and evidence of efficacy: A scoping review protocol
Source: PLoS One. 2023 May 5;18(5):e0283178. doi: 10.1371/journal.pone.0283178 (PMC10162514; doi:10.1371/journal.pone.0283178)
Supplement: S4 File — (DOCX) [file pone.0283178.s004.docx]

**S4 File. Data extraction instrument 1 - Characteristics of included studies separated by study design.**

| Study (Year) Country | Study design  Data collection | Participant description | *N* (% female) | Mean age (*SD*) | CRO elements identified |
| --- | --- | --- | --- | --- | --- |
|  |  |  |  |  |  |
|  |  |  |  |  |  |
