# Supplementary material for: Practice, governance, and culture characteristics of lived experience organisations, and evidence of efficacy: A scoping review protocol
Source: PLoS One. 2023 May 5;18(5):e0283178. doi: 10.1371/journal.pone.0283178 (PMC10162514; doi:10.1371/journal.pone.0283178)
Supplement: S6 File — (DOCX) [file pone.0283178.s006.docx]

**S6 File. Frequent CRO elements identified.**

| Study  (Year) | Culture | | | Leadership, management, & operations | | | | | | | | | | | Lived experience consumer role | | | | | | Services & support | | |
| --- | --- | --- | --- | --- | --- | --- | --- | --- | --- | --- | --- | --- | --- | --- | --- | --- | --- | --- | --- | --- | --- | --- | --- |
|  |  |  |  |  |  |  |  |  |  |  |  |  |  |  | Professional development | | | |  |  |  |  |  |
|  | Beliefs, values & principles | Social support | *Peer social interaction | Professionalization & standardization | Decision-making | Leadership | Finances | Structure | Power | Autonomy | Flexible working arrangements | Board composition | Monitor & evaluate | Membership | Mentoring & coaching | Training | Induction | Supervision | *Member engagement & inclusion | *Depth/relevance of member skill | Stakeholder relationships | Resource provision & support access | Outsourcing supports & services |
|  |  |  |  |  |  |  |  |  |  |  |  |  |  |  |  |  |  |  |  |  |  |  |  |
|  |  |  |  |  |  |  |  |  |  |  |  |  |  |  |  |  |  |  |  |  |  |  |  |
