# Supplementary material for: Practice, governance, and culture characteristics of lived experience organisations, and evidence of efficacy: A scoping review protocol
Source: PLoS One. 2023 May 5;18(5):e0283178. doi: 10.1371/journal.pone.0283178 (PMC10162514; doi:10.1371/journal.pone.0283178)
Supplement: S7 File — (DOCX) [file pone.0283178.s007.docx]

Supporting Information 7. Data extraction instrument 4 – Impact of lived experience organizations on outcomes for members of their workforce.

| Outcome 1 measure |  |  |  |  |  |
| --- | --- | --- | --- | --- | --- |
| Outcome 1 |  |  |  |  |  |
| Outcome 2 measure |  |  |  |  |  |
| Outcome 2 |  |  |  |  |  |
| Outcome 3 measure |  |  |  |  |  |
| Outcome 3 |  |  |  |  |  |
| Etc. |  |  |  |  |  |

*Note*: Outcome/s can relate to the members’ socioemotional and/or organizational outcomes.
